# Successful Chest Tube Removal Following Iatrogenic Pneumothorax in a Patient on Ventilator Support

**DOI:** 10.7759/cureus.95389

**Published:** 2025-10-25

**Authors:** Noshirwan P Gazder, Ashok Kumar, Alwina Irfan, Sania Maqbool, Faria Masood, Rawesha Hablani

**Affiliations:** 1 Department of Pulmonology, Dr. Ziauddin Hospital, Karachi, PAK; 2 Department of Hospital-Based Medicine, Dr. Ziauddin Hospital, Karachi, PAK; 3 Department of Gastroenterology, Dr. Ziauddin Hospital, Karachi, PAK

**Keywords:** chest tube management, chest tube removal, iatrogenic pneumothorax, lung injury, ventilator strategies

## Abstract

We report the case of a 14-year-old female patient who came from another hospital with complaints of a dry cough for five days, high-grade fever for three days, and altered level of consciousness for the last two days. The patient was found to be in status epilepticus, for which she was planned for intubation. During intubation, the patient was found to have desynchrony with the ventilator, with decreased breath sounds on the right side. A chest X-ray was done, which showed a right-sided pneumothorax for which a chest tube was passed. Due to prolonged hospital stay and recurrent seizures, the patient started to develop fever spikes for which the decision to remove the chest tube was taken while the patient was still on mechanical ventilation, which was successful without the recurrence of pneumothorax. Consideration should be taken that the patient had an iatrogenic pneumothorax during intubation and did not have a pneumothorax due to a lung pathology. This case emphasizes the importance of finding a balance between the timely removal of the patient's chest tube while on mechanical ventilation to prevent it from becoming a source of infection and to avoid the development of another pneumothorax with careful monitoring. The clinician's judgement, along with radiographic evidence and the patient's clinical condition, should always be strongly considered and clinically correlated.

## Introduction

A pneumothorax is a collection of air outside the lung but within the pleural cavity. It occurs when air accumulates between the parietal and visceral pleurae inside the chest. The air accumulation can apply pressure on the lung and cause it to collapse. The degree of collapse determines the clinical presentation of pneumothorax. Air can enter the pleural space by two mechanisms: either by trauma causing communication through the chest wall or from the lung by the rupture of the visceral pleura. There are two types of pneumothorax: traumatic and atraumatic. The two subtypes of atraumatic pneumothorax are primary and secondary. A primary spontaneous pneumothorax (PSP) occurs automatically without a known eliciting event, while a secondary spontaneous pneumothorax (SSP) occurs after an underlying pulmonary disease. A traumatic pneumothorax can be the result of blunt or penetrating trauma. Pneumothoraxes can be even further classified into simple, tension, or open. A simple pneumothorax does not shift the mediastinal structures, as does a tension pneumothorax. An open pneumothorax is an open wound in the chest wall through which air moves in and out [1-4].

An iatrogenic pneumothorax is a known complication of invasive procedures such as pulmonary needle biopsy (transthoracic and transbronchial), placement of a central venous line, or positive pressure ventilation [5]. The traditional treatment for pneumothorax in mechanically ventilated patients has been chest tube thoracostomy [6]. Pneumothorax secondary to barotrauma, tension pneumothorax, and concurrent septic shock are all significant independent risk factors for mortality in critically ill patients [7]. In terms of iatrogenic pneumothorax, the mortality rate of patients with ventilator-related pneumothorax was significantly higher than that of patients with procedure-related pneumothorax [8].

Here, we report the case of an iatrogenic pneumothorax in a 14-year-old patient who came with status epilepticus whose chest tube was subsequently removed while the patient was still on ventilator support.

## Case presentation

A 14-year-old female patient was referred to the emergency department of our hospital in early 2025 with chief complaints of dry cough for five days, sore throat, and fever documented at 101-103°F for three days, along with an altered level of consciousness for the last two days. She had a history of four days of hospitalization with similar complaints in another hospital, but was referred out due to a lack of facilities. Upon arrival in the emergency department, the patient was ill-looking, drowsy, and not arousable with a Glasgow Coma Scale (GCS) score of 9/15 (eye: 4 points, verbal: 2 points, motor: 3 points). Her vitals were as follows: blood pressure 108/59 mmHg, pulse 119 beats/min, regular with normal volume, and respiratory rate 17 breaths/min. She had no signs of jaundice, anemia, clubbing, cyanosis, or edema on general physical examination. Chest examination revealed decreased air entry on the right side.

**Figure 1:**
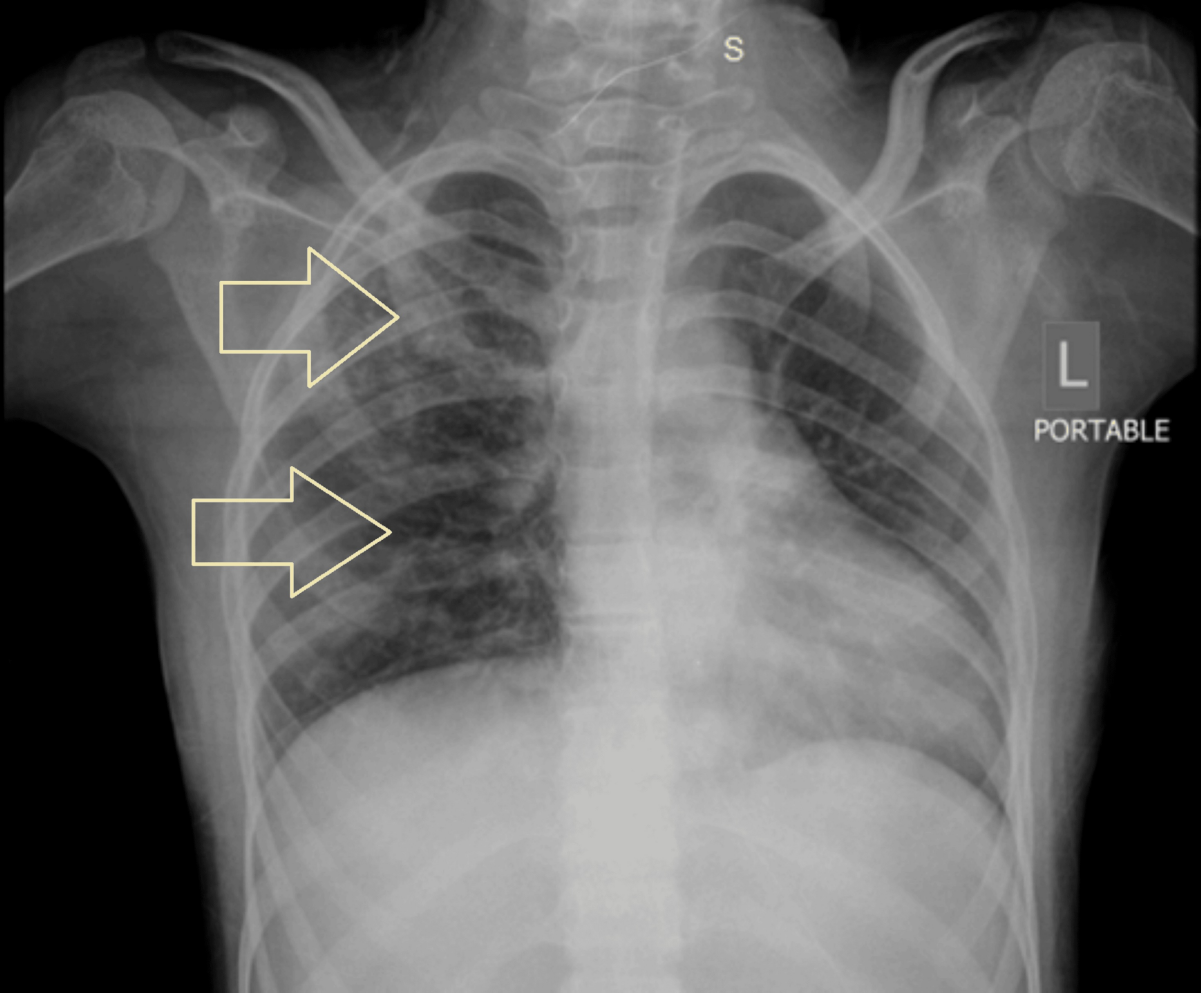
Chest X-ray at the time of admission showing ground-glass haze with infiltrates identified in the right upper and mid zones (white arrow)

Laboratory examination at the time of admission revealed the following: hemoglobin 10.1 gm/dl, hematocrit 31%, total leukocyte count 11.2×10⁹, neutrophil 75%, lymphocyte 14%, platelet count 462×10⁹, serum sodium 145 mEq/L, potassium 3.1 mEq/L, chloride 105 mEq/L, bicarbonate 28.1 mEq/L, urea 9 mg/dl, creatinine 0.30 mg/dl, and procalcitonin 0.466 ng/ml. Chest X-ray at the time of admission showed ground-glass haze with infiltrates identified in the right upper and mid zones; both costophrenic angles and both domes of the diaphragm appeared normal (Figure 1).

**Figure 2:**
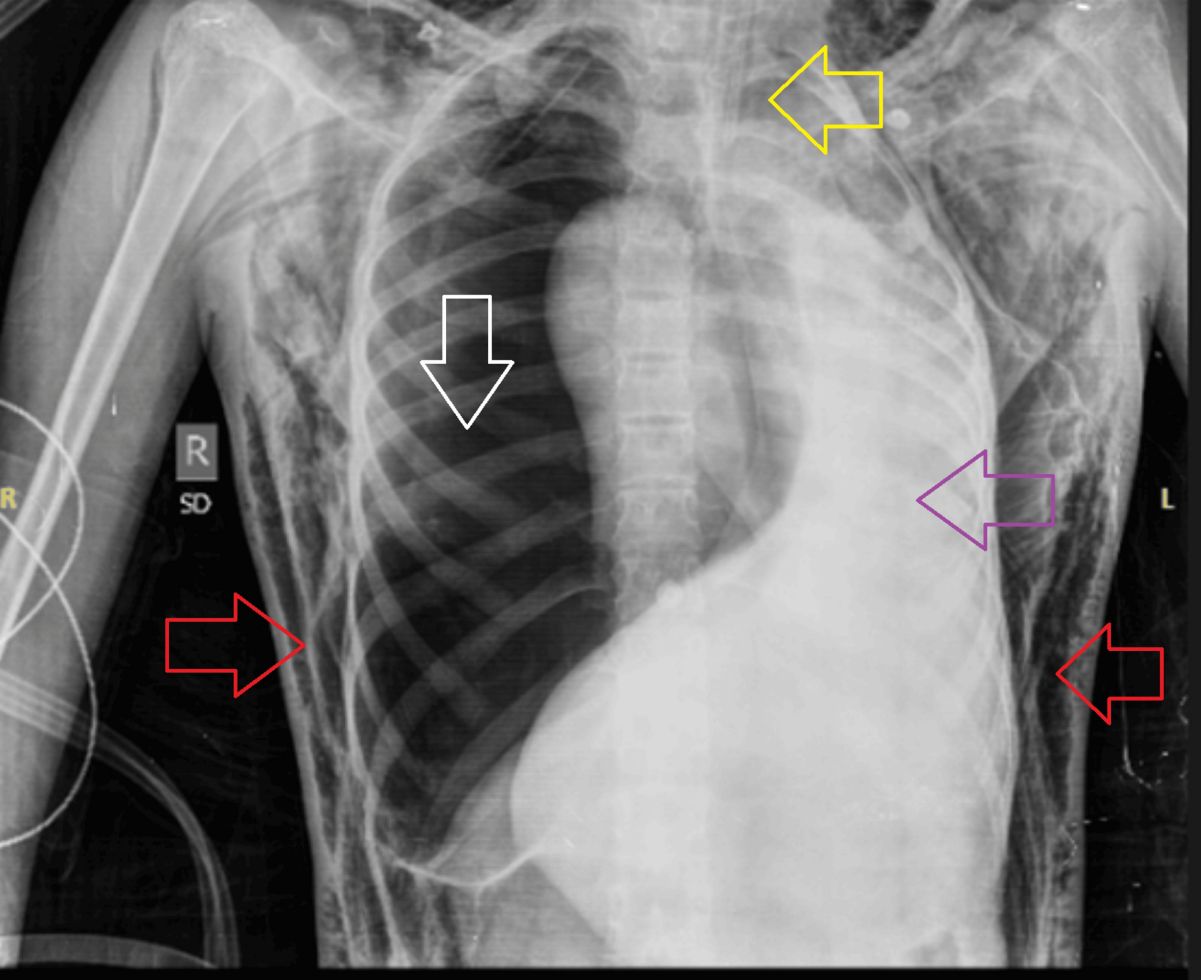
Right hemithorax demonstrating the absence of vascular markings (white arrow) with contralateral mediastinal shift (purple arrow), complete collapse of the right lung, and extensive subcutaneous emphysema along both chest walls (red arrow), with the endotracheal tube seen in situ (yellow arrow)

The patient was admitted to the medical intensive care unit with the impression of meningitis, complicated pneumonia, and/or neuroleptic malignant syndrome. Magnetic resonance imaging (MRI) of the brain was done, which showed both cerebellar hemispheres, including cerebral tonsils and vermis, showing disorganized cerebellar folia with vertical orientation, white matter arborization, and cortical hypertrophy, giving mild high signals on fluid-attenuated inversion recovery (FLAIR). Focal areas of T2 hyperintensities were appreciated at the subcortical bilateral frontal and parietal locations, suggesting focal areas of ischemic demyelination. No other abnormal post-contrast brain parenchymal or meningeal enhancement was seen. Neurology opinion was taken, who advised for an electroencephalogram (EEG), which revealed the patient was in status epilepticus, as the EEG showed the presence of a continuous sharp and fast rhythmic activity, with episodes of left-sided facial twitching along with gaze deviation and light body jerking. The patient's seizure activity was not controlled by anti-epileptics, and she was planned to be intubated and started on midazolam infusion. An endotracheal tube (ETT) of size 7.5 mm was passed in the patient, but upon passing the ETT, she was seen to be desaturating. Upon attaching to the ventilator support, the patient was not generating tidal volumes. The ventilator was detached, and manual ventilation using ambu bagging was started to maintain O₂ saturation. During ambu bagging, there was resistance felt; chest auscultation revealed decreased breath sounds on the right hemithorax. An urgent chest radiograph demonstrated an ETT in situ. The right hemithorax appeared hyperlucent with an absence of vascular markings, accompanied by a contralateral mediastinal shift and complete collapse of the right lung. Extensive subcutaneous emphysema was noted along both chest walls and in the visualized soft tissues of the neck. Additionally, diffuse haziness was observed in the left lung field, likely representing congestive changes (Figure 2). 

**Figure 3:**
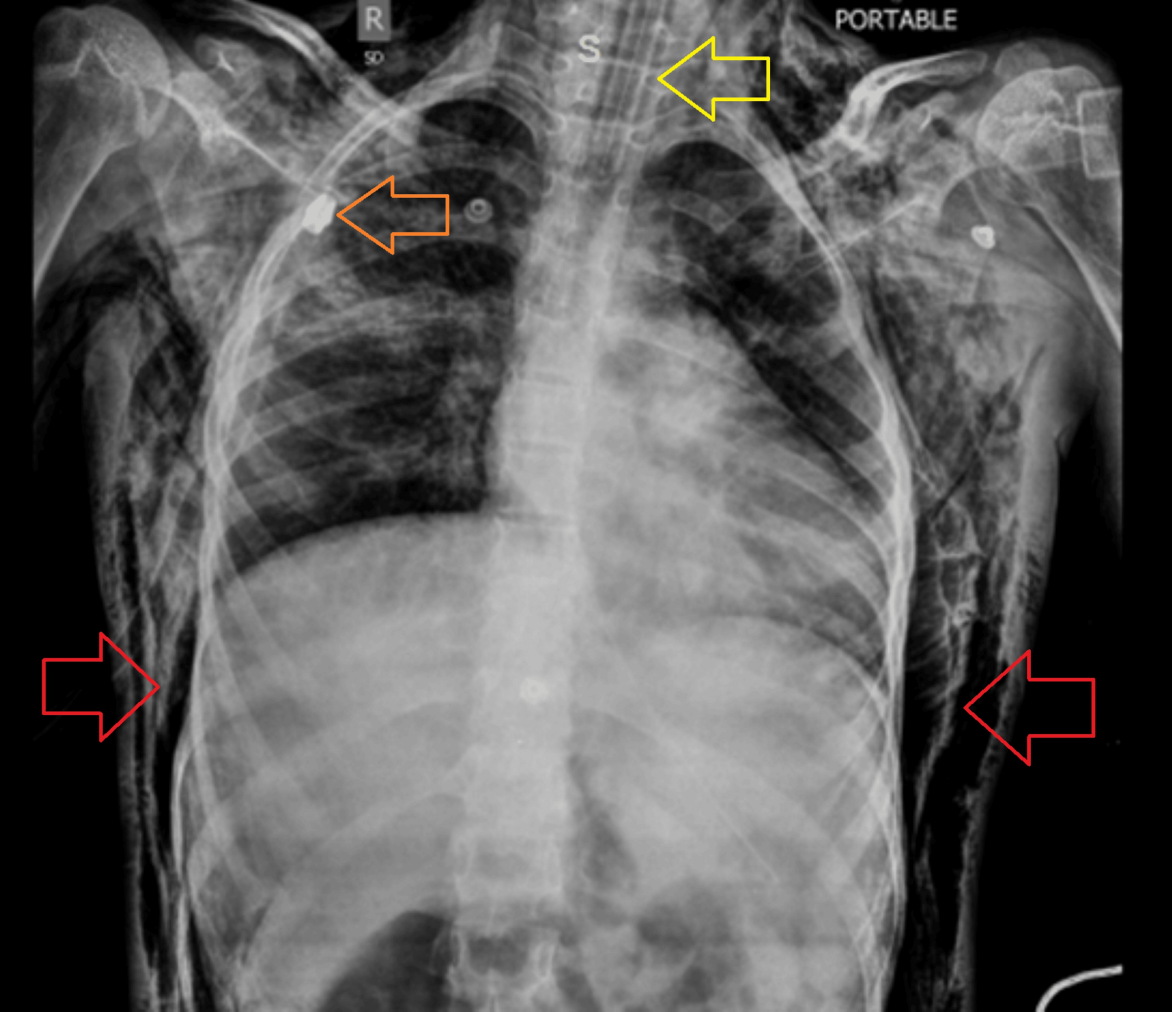
Chest X-ray immediately after passing the chest tube (orange arrow) along with bilateral subcutaneous emphysema along the chest wall (red arrow) with the endotracheal tube in place (yellow arrow)

Following this, tube thoracostomy was urgently performed with a 24-French (Fr) chest tube in the right fourth intercostal space in the mid-axillary line under aseptic conditions. A gush of air was observed after pleural puncture, and the tube was attached to a chest drain. A repeat chest radiograph obtained after tube thoracostomy demonstrated the interval placement of a right-sided chest tube with improved aeration of the right lung. Prominent interstitial markings and haziness were noted in the right lung, along with extensive subcutaneous emphysema involving both chest walls and the visualized soft tissues of the neck and abdomen. The ETT remained in situ. An inhomogeneous area of airspace opacification was again observed in the right mid zone, suggestive of a pulmonary infection (Figure 3).

The patient was kept on Synchronized Mandatory Volume Control Ventilation (SCMV) mode of the ventilator, with a respiratory rate (R/R) of 18 breaths/min, a fraction of inspired oxygen (FiO₂) of 40%, a positive end-expiratory pressure (PEEP) of 5 cm of water, an inspiratory tidal volume (VTi) of 400 ml, and an end-tidal volume (Vte) of 380-400 ml. She had a bronchoscopy done on the same day, from which a relevant workup was sent for analysis. The patient's Pneumonia Plus Panel detected *Klebsiella pneumoniae*, and appropriate antibiotics were started after reviewing antimicrobial resistance genes.

**Figure 4:**
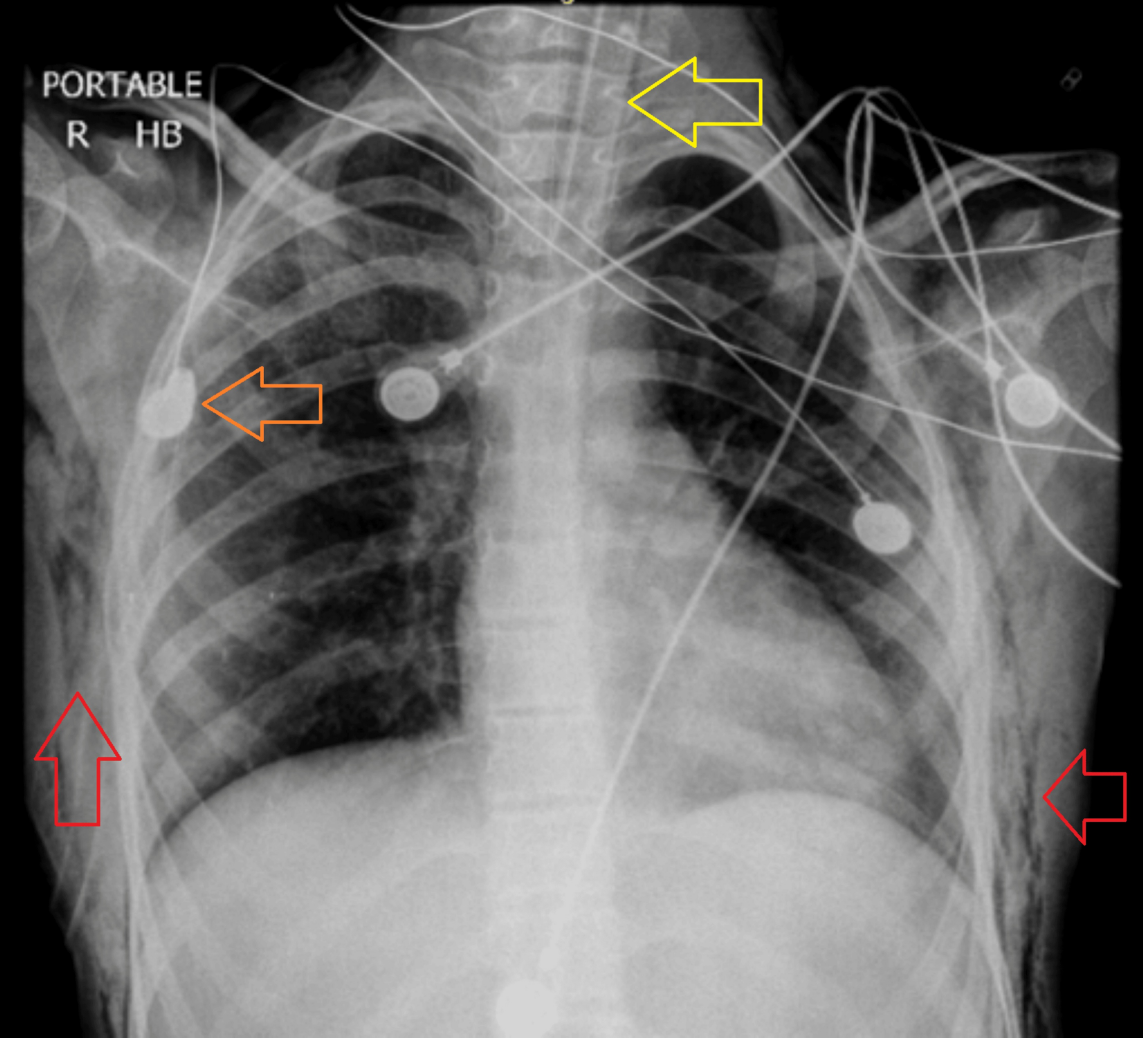
Chest X-ray taken on the day after passing the patient's chest tube (orange arrow) with the endotracheal tube in place (yellow arrow) and bilateral subcutaneous emphysema (red arrow)

The following day, the patient's FiO₂ was tapered down to 21%, and anti-epileptics as well as sedation were continued as recommended by the neurology team. A chest radiograph obtained one day after chest tube placement (Figure 4) showed the re-demonstration of the right-sided chest tube and ETT. Subcutaneous emphysema along both chest walls and the visualized soft tissues of the neck and abdomen was noted, with interval reduction compared to the previous chest radiograph. The inhomogeneous area of airspace opacification in the right mid zone persisted, suggestive of pulmonary infection. Due to the patient having persistent seizures, ventilator support had to be continued along with sedation (midazolam infusion) and multiple anti-epileptics.

On the eighth day of hospitalization, the patient started to develop fever spikes, prompting the decision to change the patient's lines. Her central venous catheter (CVC) and Foley's catheter were changed. To remove any further source of fever, the decision to remove the patient's chest tube was also taken. As per recommendations, a chest tube is not ideally removed if a patient has a fever. As serial chest radiographs demonstrated the complete resolution of the pneumothorax with full re-expansion of the lung, absence of air leak from the chest drain, and resolution of subcutaneous emphysema, the decision was made to remove the chest tube. After a discussion with other senior faculty members, it was decided to clamp the patient's chest tube and then to repeat the patient's chest X-ray after six hours.

**Figure 5:**
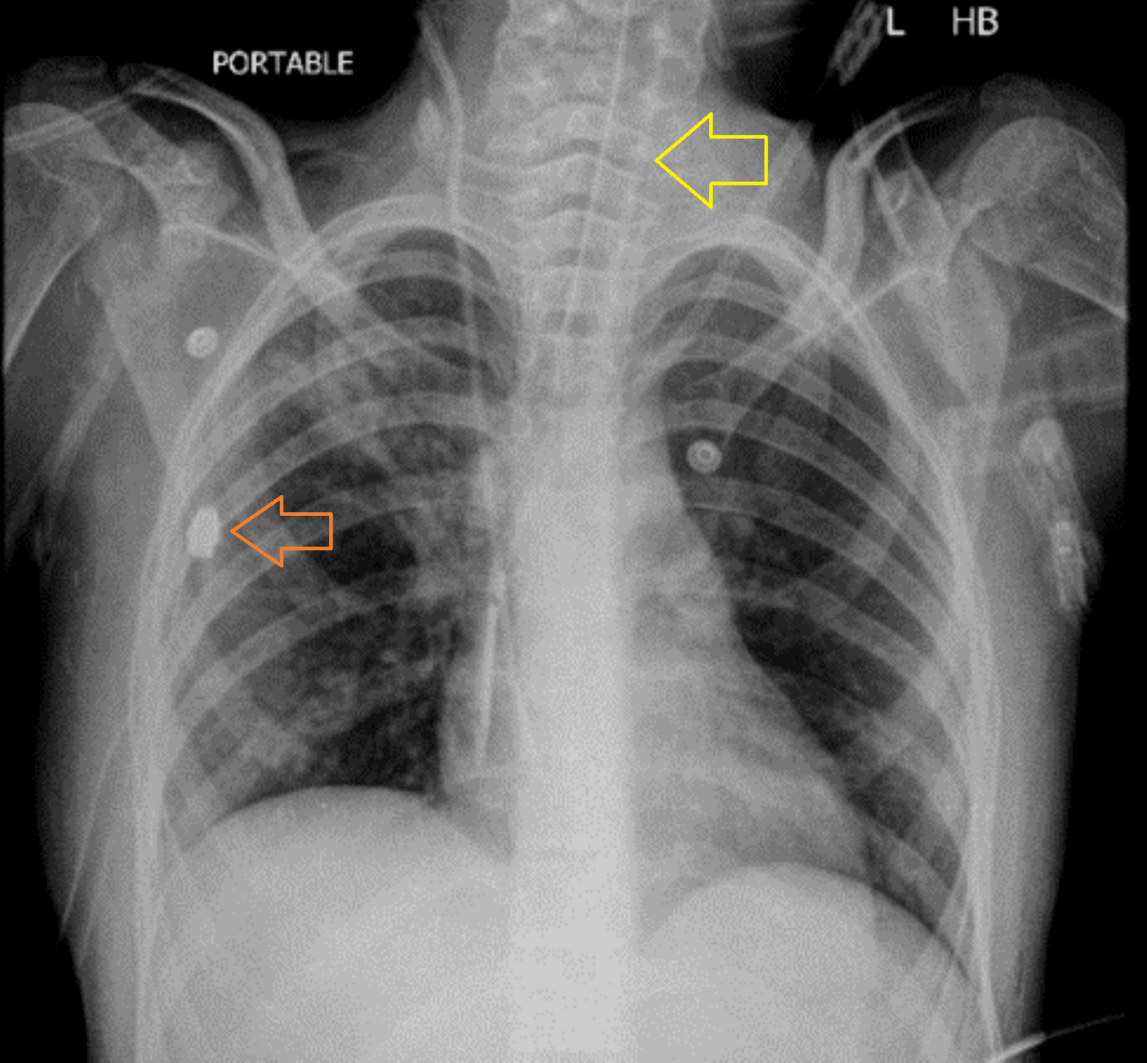
Chest X-ray on the morning of the eighth day of hospitalization with the chest tube (orange arrow) and endotracheal tube (yellow arrow) in place

**Figure 6:**
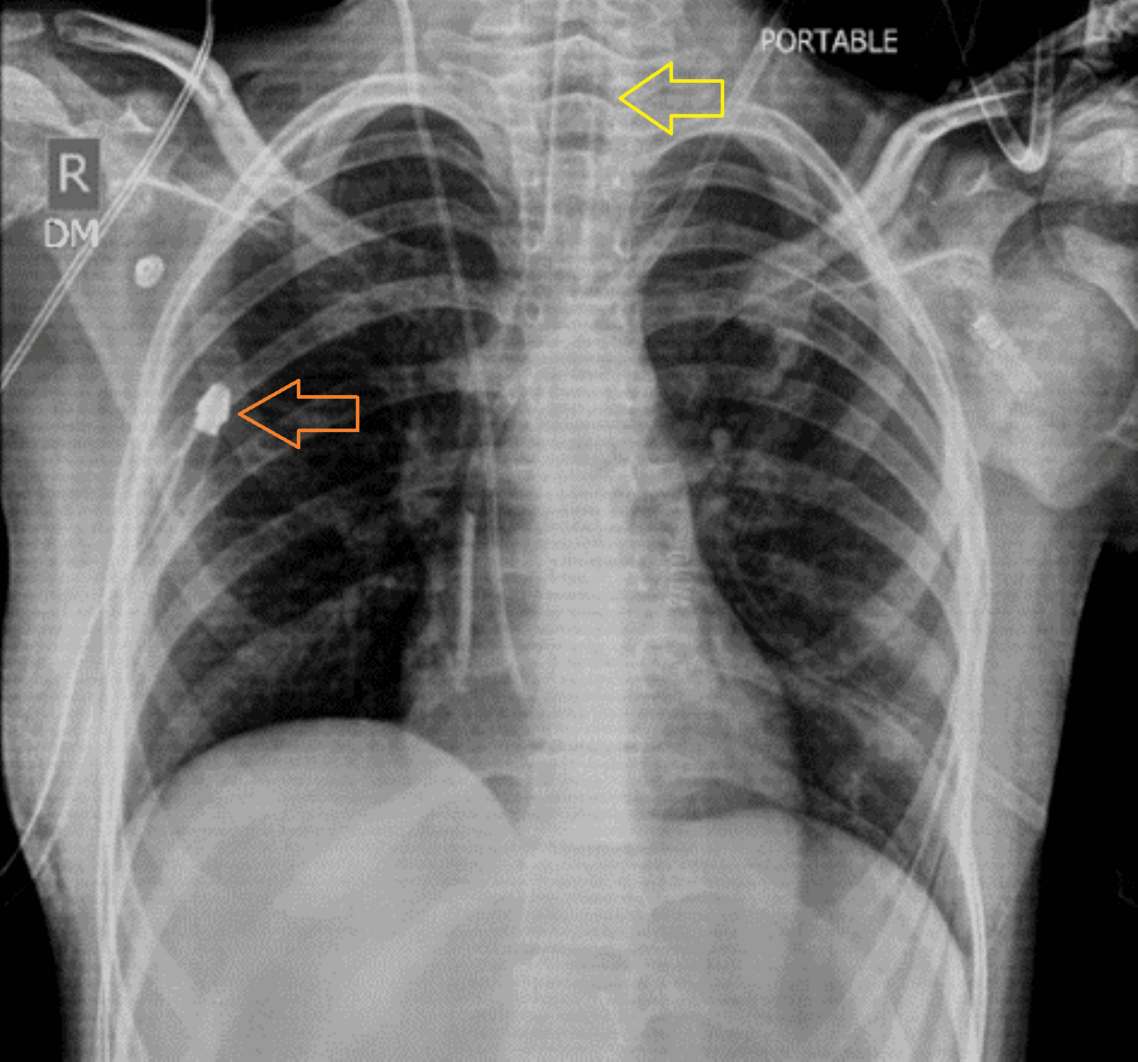
Chest X-ray on the eighth day of hospitalization, six hours after clamping the chest tube, with the chest tube (orange arrow) and endotracheal tube (yellow arrow) in place

On the eighth day of hospitalization, the patient's chest tube was clamped at 12:30 pm, and she was on SCMV mode of the ventilator, with an R/R of 16 breaths/min, an FiO₂ of 21%, a PEEP of 5 cm of water, a VTi of 400 ml, and a Vte of 380-400 ml. X-rays are shown in Figure 5 and Figure 6, respectively. The chest X-ray report after comparing with the morning chest X-ray showed the re-demonstration of ETT, central venous pressure (CVP) line, and right-sided chest tube. No definite patch of consolidation/collapse was seen in the left lung field. The rest of the findings were grossly unchanged since the previous X-ray.

**Figure 7:**
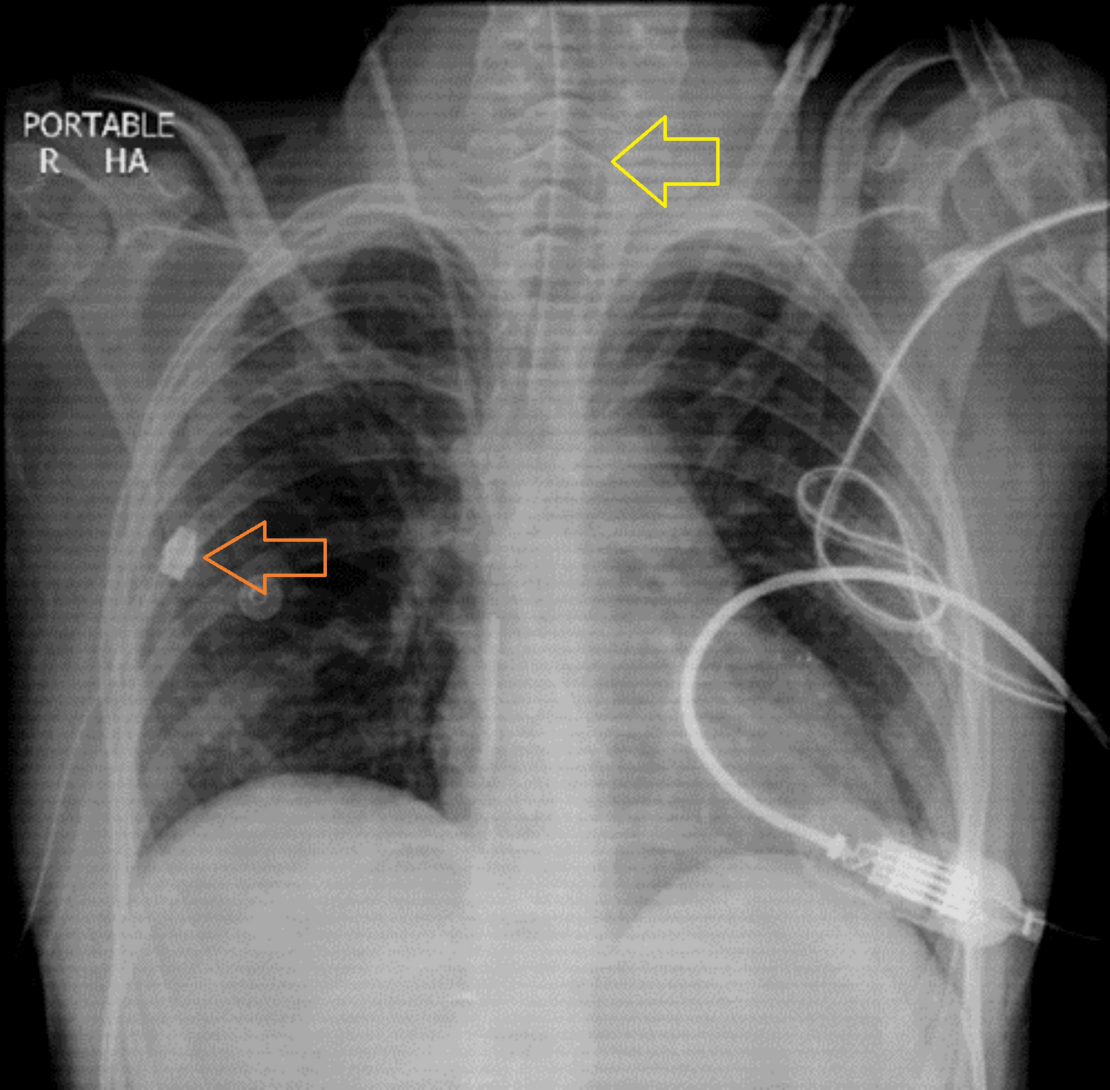
Chest X-ray taken on the ninth day of hospitalization, after clamping the chest tube for 24 hours, with the chest tube (orange arrow) and endotracheal tube (yellow arrow) in place

The chest tube was clamped for a further 24 hours, and a chest X-ray was repeated the next day on the ninth day of hospitalization (Figure 7).

The report showed the re-demonstration of the ETT, CVP line, and right-sided chest tube. No definite patch of consolidation/collapse was seen in the left lung field. The rest of the findings were grossly unchanged since the previous X-ray. The patient's ventilator settings remained unchanged from the previous day.

**Figure 8:**
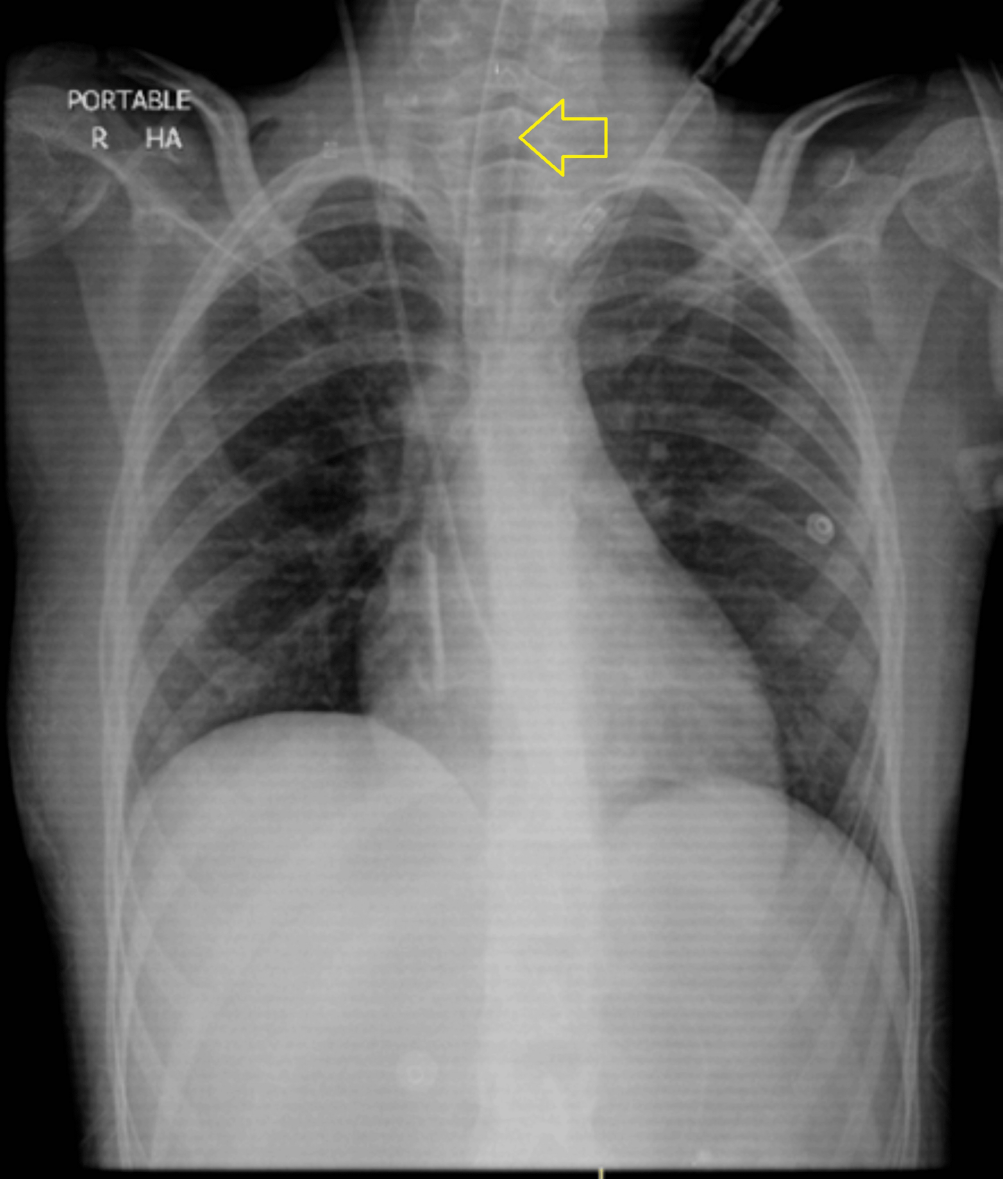
Chest X-ray post-chest tube removal with the endotracheal tube in place (yellow arrow)

The patient remained clinically and vitally stable after the removal of the chest tube with the same ventilator settings as the previous few days. The patient's post-chest tube removal chest X-ray is shown in Figure 8.

The chest X-ray was reported as the re-demonstration of the ETT and CVP line. Interval removal of the right-sided chest tube was noted. No definite patch of consolidation/collapse was seen in either lung field. The rest of the findings were grossly unchanged since the previous X-ray.

Unfortunately, the patient was still having frequent seizure-like activity on the EEG, prompting the need for the continued use of midazolam infusion and prolonged intubation. Ultimately, due to prolonged intubation, she underwent a tracheostomy. Because of the extended stay of the patient due to her condition, she again started to develop fever spikes. A high-resolution computed tomography (HRCT) of the chest was done on the 18th day of admission once the patient could be safely moved, which showed an irregular patch of consolidation with adjacent nodular infiltrates identified in the apicoposterior segment of the right upper lobe. A similar patch of consolidative atelectasis was identified in the medial basal segment of the lower lobe with adjacent fibrotic bands as well. Findings were suggestive of pulmonary infection. Minimal pleural effusion was identified on the left side. There was no pleural effusion or pneumothorax on the right side. Her heart and pericardium appeared normal. The major upper airways appeared patent. Gradually, the patient's EEG settled with a reduction in epileptiform discharges, the need for midazolam infusion settled, and she was gradually weaned off ventilator support and was stepped down to the high-dependency unit (HDU). The patient would have intermittent bouts of jerky movement, prompting the need to carry out repeat EEGs and further need to adjust her anti-epileptic medications. She was eventually discharged 57 days after admission, once she was completely seizure-free on the decided regimen of anti-epileptics by the neurologist. The patient was discharged with a GCS score of 10/10T (eye: 4 points, motor: 6 points, V1T: verbal response not testable due to tracheostomy). The patient's tracheostomy was reversed around two months after her discharge. Anti-epileptic medications have been reduced, and she is currently on three anti-epileptics with a plan for further tapering by the neurologist on further outpatient department (OPD) visits. 

## Discussion

A patient with iatrogenic pneumothorax who is on mechanical ventilation is never a routine problem; it shows up with sky-high stakes and a scant warning [8]. The incident mentioned here, pulling out a chest tube on an adolescent who is sedated, became prominent since the patient appeared stable and no air leak was witnessed at that time. Such silent events call for a team that can assess the moment carefully, barotrauma or not.

Most clinicians state that a ventilator-associated pneumothorax usually traces back to raw tears or overinflated alveoli inflicted by continuous positive pressure (CPP). Statistics show a higher mortality and morbidity rate linked to it [9]. In this clinical illustration, the air pocket probably burst loose during the rush for intubation. Before the monitor alarms could catch up, an unusual stiffness in the bag-mask circuit signaled, and immediate oxygen desaturation occurred. Pressure in the pleural cavity soon paced ahead of the body's systemic blood pressure, turning the pocket into a classical tension pneumothorax. In a matter of minutes, the subcutaneous air ballooned across the upper chest and neck, making tube thoracostomy a non-negotiable, life-saving measure.

Conventionally, interventionists wait till a pneumothorax resolves completely, so the lungs can fully re-expand and there are no air leaks for an ample period of time before the chest tube is pulled out [10]. This caution is usually applied to patients on positive pressure ventilation, where even a small hidden leak can masquerade as safety, while premature removals may trigger the immediate return of a pneumothorax [11]. The fact is that most international guidelines concede that tubes in uncommunicative or deeply sedated patients are held for many days or even weeks, indicating a widespread dread of recurrence [12].

By contrast, this case report focuses on devising a protocol to determine whether the chest tube was essential or not. For the first six hours, clinicians resorted to a methodical clamping trial and later to a full 24-hour one; imaging was done for each interval to assess relapse. Despite the patient being on synchronized controlled ventilation with a stable PEEP throughout, no air leaks were revealed in follow-up X-rays. This degree of confidence allowed the safe removal of the chest drain, while the patient exhibited no clinical decline and neither any further radiographic changes.

The case at hand prompts an essential reevaluation of the time period a chest drain should be kept for a patient on mechanical ventilation. Recent studies suggest that once the air leak is sealed and the lungs are clear, the tube can be removed earlier than tradition dictates, even for ventilated patients [13]. Keeping the drain in for a longer period invites infections, restricts patient mobilization (as the patient has multiple lines attached), and increases nursing time [14].

The case became complicated due to an established *Klebsiella pneumoniae* infection, which carries resistance genes, less responsive to many antibiotics. This nosocomial germ is capable of putrefying around foreign tubes easily; this means that with every minute that the tube stays in, it provides a greater window for the pathogen to grow and spread [15]. Ultimately, early chest tube retraction becomes an infection control strategy in order to counter busy and high-pressure situations in the intensive care unit.

This case depicts an example of how to treat pneumothorax caused by mechanical ventilation, which relies on evidence and personalized judgment [8]. With vigilant monitoring, sequential radio imaging, and multidisciplinary team collaborations, it is possible that clinicians may remove the chest tube earlier, despite the patient being sedated or neurologically weak [16]. Such measures can lower the chances of infections as well as procedural complications, while ensuring patient safety, though larger prospective trials might be required to confirm this approach.

## Conclusions

This case demonstrates that removal of a chest tube while a patient is on mechanical ventilation is considered safe. Consideration should be taken that the patient had an iatrogenic pneumothorax during intubation and did not have a pneumothorax due to a lung pathology. The patient's chest tube was removed to eliminate any source of further infection in a patient whose course of hospital stay was predicted to be prolonged. The clinician's judgement, along with radiographic evidence and the patient's clinical condition, should always be strongly considered and clinically correlated. This case further emphasizes the importance of finding a balance between the timely removal of the patient's chest tube while on mechanical ventilation to prevent it from becoming a source of infection and to avoid the development of another pneumothorax with careful monitoring.
